# Protein Tyrosine Phosphatase 1B Inhibitors of *Pueraria lobata* Based on the Spectrum–Effect Relationship by Q-Marker Selection

**DOI:** 10.3390/molecules29122731

**Published:** 2024-06-08

**Authors:** Yong Zhang, Haipeng Liu, Tianci Lv, Mengqian Xiao, Guihua Gao

**Affiliations:** 1 School of Pharmacy, Jining Medical University, Rizhao 276826, China; zhang_2211@163.com; 2 School of Pharmacy, Shandong University of Traditional Chinese Medicine, Jinan 250355, China; liuhaipeng1005@163.com (H.L.); ays51314@163.com (T.L.); 15954112102@163.com (M.X.)

**Keywords:** PTP1B, inhibitory activity, *Pueraria lobata*, spectrum–effect relationship

## Abstract

*Pueraria lobata* (*P. lobata*), a traditional anti-diabetic medicine mainly composed of flavonoids and isoflavones, has a long history in diabetes treatment in China. However, the anti-diabetic active component is still unclear. Recently, protein tyrosine phosphatase 1B (PTP1B) has been a hot therapeutic target by negatively regulating insulin signaling pathways. In this study, the spectrum–effect relationship analysis method was first used to identify the active components of *P. lobata* that inhibit PTP1B. The fingerprints of 12 batches of samples were established using high-performance liquid chromatography (HPLC), and sixty common peaks were identified. Meanwhile, twelve components were identified by a comparison with the standards. The inhibition of PTP1B activity was studied in vitro by using the p-nitrophenol method, and the partial least squares discriminant analysis, grey relational analysis, bivariate correlation analysis, and cluster analysis were used to analyze the bioactive compounds in *P. lobata*. Peaks 6, 9 (glycitin), 11 (genistin), 12 (4′-methoxypuerarin), 25, 34, 35, 36, 53, and 59 were considered as potentially active substances that inhibit PTP1B. The in vitro PTP1B inhibitory activity was confirmed by glycitin, genistin, and 4′-methoxypuerarin. The IC_50_s of the three compounds were 10.56 ± 0.42 μg/mL, 16.46 ± 0.29 μg/mL, and 9.336 ± 0.56 μg/mL, respectively, indicating the obvious PTP1B inhibitory activity. In brief, we established an effective method to identify PTP1B enzyme inhibitors in *P. lobata*, which is helpful in clarifying the material basis of *P. lobata* on diabetes. Additionally, it is evident that the spectrum–effect relationship method serves as an efficient approach for identifying active compounds, and this study can also serve as a reference for screening bioactive constituents in traditional Chinese medicine.

## 1. Introduction

Diabetes mellitus (DM) is the collective name for a group of metabolic diseases characterized by hyperglycemia, which is a major challenge to public health due to the high incidence and low cure rate [1]. According to the report of the International Diabetes Federation (IDF), the global prevalence of diabetes among adults aged 20–79 increased from 4.6% to 10.5% of the worldwide population from 2000 to 2021. More seriously, global morbidity will sharply increase to 11.3% by 2030 and 12.2% by 2045, respectively. The prevalence of diabetes has also imposed a substantial economic burden on nations and families, with the global healthcare expenditure for individuals aged 20–79 escalating from USD 232 billion in 2007 to USD 966 billion in 2021 [2]. Type 2 DM (T2DM) is the most common type of DM, accounting for approximately 90% of cases [3]. The main pathogeneses of T2DM are insulin secretion defects and insulin resistance. Insulin is a protein hormone synthesized by pancreatic β-cells and secreted after stimulation. It is the only hormone that can promote the synthesis of glycogen, fat, and protein while reducing blood glucose in the body. Its main physiological role is to act on tissues, such as the liver, muscle, and fat, to regulate metabolism [4].

**Figure 1:**
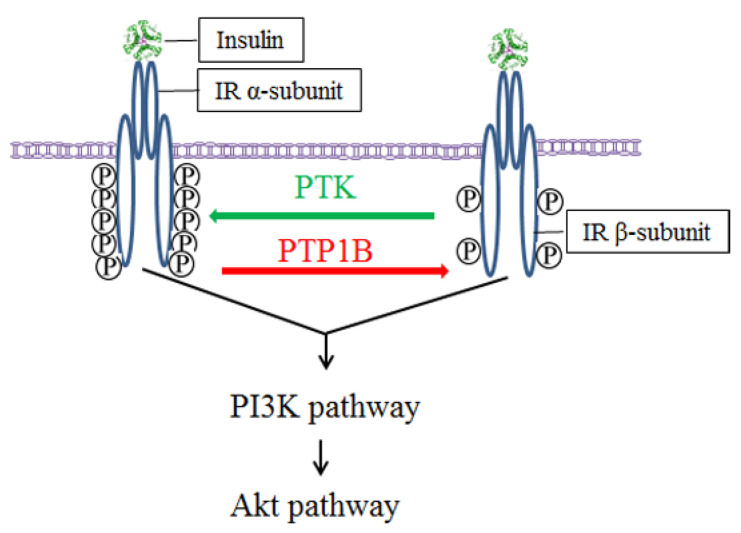
Mechanisms of TPK and PTP1B regulating insulin signaling. Green arrow represents phosphorylation; red arrow represents dephosphorylation.

As shown in Figure 1, the insulin signaling cascade is highly regulated by two key classes of enzymes: protein tyrosine kinases (PTKs) and protein tyrosine phosphatases (PTPs). Insulin binds to the α-subunit of the insulin receptor, thereby activating the tyrosine kinase activity of the β-subunit located in the cell and the phosphorylation of specific tyrosine residues catalyzed by PTK. Then, the phosphorylation of the tyrosine residues of downstream signaling proteins (such as insulin receptor substrates) is initiated, thereby initiating intracellular signal transduction [5] and activating the transduction of the PI3K-Akt pathway. Thus, glucose is taken up by the cells from the blood [6], resulting in the inhibition of glycogen synthesis and, thus, lowering the blood glucose.

Protein tyrosine phosphatase 1B (PTP1B), an intracellular PTP and member of the NT1 subfamily, has a total length of approximately 50 kDa and is located in the endoplasmic reticulum. The PTP domain is oriented in the cytoplasm due to hydrophobic stretching at the C-terminal. PTP1B dephosphorylates activated insulin receptors and insulin receptor substrates and is, therefore, a key enzyme in the cellular regulation of insulin signaling. Only the phosphorylated protein can exert the biological effect of lowering blood glucose. Phosphorylation usually occurs at the tyrosine (Tyr), serine (Ser), and threonine (Thr) residues of proteins [7,8]. The maintenance of a dynamic balance between phosphorylation and dephosphorylation is essential for the regulation of signaling pathways and the preservation of glucose homeostasis [9]. 

The phosphorylation level is key to glucose metabolism in the insulin signaling pathway. Insulin stimulates Tyr66 phosphorylation in the PTP1B catalytic region, which, in turn, activates PTP1B to dephosphorylate insulin receptors and their substrates, thereby negatively regulating insulin-promoted glucose metabolism. Physiologically, PTKs and PTPs collaborate to maintain the homeostasis of the insulin signaling pathway [10]. However, when PTP1B is overexpressed in vivo, phosphorylated insulin receptors are restored to their non-phosphorylated form within minutes [11]. Long-term preservation of low phosphorylation levels of insulin receptors is one of the causes of insulin resistance [6]. In T2DM individuals, PTP1B is overexpressed [12], resulting in the insufficient phosphorylation of insulin receptors, thereby impairing the normal regulatory function of the insulin signaling pathway. The change in the biological activity of insulin in the tissue prompts an increase in insulin secretion by pancreas islet β-cells, counteracting the lack of insulin action and resulting in hyperinsulinemia. As the disease progresses, pancreas islet β-cells begin to fail, and a vicious cycle ensues, leading to further a decline in insulin secretion and the worsening of hyperglycemia. Knockout of PTP1B promotes the phosphorylation of insulin receptors in mice, leading to the enhancement of glucose metabolism [13] and the decrease of glucose and insulin in the blood [14]. Compared to PTP1B+/+ mice, PTP1B−/− mice show increased insulin sensitivity in the liver and muscle and insulin resistance induced by a high-fat diet [15,16]. Therefore, PTP1B is an important target for the treatment of insulin resistance, and PTP1B inhibitors have potential for the treatment of T2DM.

In a clinic, T2DM is generally treated by administering hypoglycemic and lipid-lowering medications and supplemented by controlling dietary regimen and engaging in moderate physical activity [17,18]. In recent decades, natural products have emerged as valuable bioactive resources for the development of anti-T2DM medications. Traditional Chinese medicines (TCM) have been long-term used for the treatment of T2DM. For example, Astragalus, Bupleurum, *Pueraria lobata* (*P. lobata*), Poria cocos, Atractylodes, Codonopsis pilosula, and Scutellaria baicalensis are commonly used in clinics for the therapy of T2DM [19]. Gegen Qinlian Decoction, composed of *P. lobata*, Scutellaria baicalensis, Coptis, and Licorice, is beneficial for the regulation of pancreas islet conduction, inflammatory signaling transduction, oxidative stress, and intestinal flora, thereby improving insulin resistance [20]. 

In TCM, *P. lobata* is one of the most used herbal drugs for the treatment of DM. Approximately 35 species of *P. lobata* are known worldwide, mainly distributed in the tropical and subtropical regions of East and Southeast Asia [21], north to China, Japan, south to Malaysia, west to India, Sri Lanka, and east to Indonesia. There are eight species and two varieties in China. Both *Puerariae lobatae radix* and *Puerariae thomsonii radix* are included in the Pharmacopoeia of the People’s Republic of China. 

*P. lobatae*, the dried root of *P. lobata* (Willd.) Ohwi, is a kind of TCM used as both medicine and food. Its components include flavonoids [22], isoflavones [23], pentacyclic triterpenoids, triterpenoid saponins, coumarins, Pueraria glycosides, and polysaccharides [24,25]. The modern pharmacological studies found various bioactivities, such as anti-inflammation [26], anti-atherosclerosis [27], anti-hyperlipidemia [28], anti-hypertension [29] and liver protection [30]. More and more research has indicated that *P. lobata* exerts anti-diabetic effects by reducing insulin resistance, increasing insulin release, and improving insulin sensitivity, enhancing glucose uptake and metabolism [31,32]. In detail, puerarin, the main component of *P. lobata*, exerted an anti-diabetic effect by inhibiting the TGF-β1/Smad2 pathway in streptozotocin-induced mice [33]. A 75% butanol fraction of *P. lobata* extract inhibited PTP1B activity to enhance the glucose uptake capacity in insulin-resistant HepG2 cells and improve glucose control and tolerance in diabetic mice [34]. Moreover, Genistein, another of the main components of *P. lobata*, has a reversible effect on insulin release [15]. However, the effective components of *P. lobata* for the therapy of T2DM have not been fully demonstrated.

Spectrum–effect relationship analysis uses powerful mathematical and statistical methods, such as grey relational analysis (GRA), correlation analysis (CA), and partial least squares discrimination analysis (PLS-DA), to study the relationship between fingerprint data and pharmacodynamic indicators, to clarify the material basis of the pharmacological action of TCM, and to analyze the bioactive substances in fingerprints [35]. However, there is a lack of reports regarding the spectrum–effect relationship analysis between *P. lobata* and its inhibition of PTP1B. In the present study, the spectrum–effect relationship method was proposed to study the components of *P. lobata* on inhibiting PTP1B activities and aimed to explore the material basis of the hypoglycemic mechanism of *P. lobata*.

## 2. Results and Discussion 

### 2.1. Establishment of Fingerprint of P. lobata Extract

The HPLC column plays a crucial role in HPLC analysis, and it is also very important to the establishment of TCM chromatogram fingerprints. Therefore, following a comparison of the Tskgel ODS-100V C18 (Tosoh, Tokyo, Japan) (4.6 mm× 250 mm, 3 μm) and the ZORBAX SBC18 RP (Agilent Technologies, Santa Clara, CA, USA) (4.6 mm × 250 mm, 5 μm) columns, the Tskgel ODS-100V column was selected for the separation of compounds from the *P. lobata* extract, as it had a better peak-to-peak resolution. Moreover, the number of peaks in the chromatogram obtained using this column was the highest, at 254 nm.

**Figure 2:**
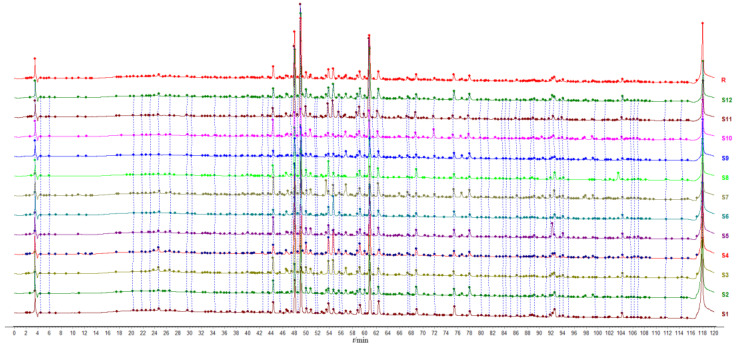
HPLC fingerprint chromatograms of *P. lobata*.

Under optimized chromatographic conditions, the HPLC fingerprints of the *P. lobata* samples were arranged neatly, and the reference fingerprints were generated using the Similarity Evaluation System for Chromatographic Fingerprint of TCM, shown in Figure 2. In the fingerprints, the peak for glycitein (peak 20) was selected as the reference peak for calculating the relative retention time and relative peak area of each common peak because of its moderate size and good separation from other chromatographic peaks. As a result, 60 characteristic peaks were identified, which accounted for over 80% of the total peak areas in the fingerprint. A similarity analysis of the chromatographic profiles of all the samples revealed that all the similarity values between the fingerprints of the different batches of *P. lobata* and the reference fingerprints exceeded 0.80, with a range of 0.888 to 0.967 (Table 1). These results demonstrated minimal chemical variation among samples in the major producing regions, thereby confirming the reliability of the established fingerprint. However, the similarity values between S11 and S1, S2, S3, S5, S6, S7, S9, and S10 were 0.735, 0.727, 0.810, 0.856, 0.757, 0.842, 0.796, and 0.823, respectively, indicating that the contents of the chemical components of S11 were different from those of the other samples. The results demonstrated that the production area had a significant influence on the chemical composition and content of *P. lobata*.

Moreover, 12 reference standards, including 3′-hydroxypuerarin, puerarin 6″-*O*-xyloside, 3′-methoxypuerarin, glycitin, genistin, 4′-methoxypuerarin, ononin, daidzein, glycitein, genistein, formononetin, and biochanin A, were dissolved in methanol to prepare a pure reference standard solution and a mixed reference standard solution. They were then injected into the HPLC system, and the retention time of each reference standard was recorded for qualitative analysis. Twelve peaks of fingerprints were identified by comparing their retention times. Chromatograms of the reference fingerprints and the mixed reference standard solution are shown in Figure S1.

To validate the HPLC method, one sample (S4) was randomly selected for precision, repeatability, and stability validation tests. Precision was evaluated using six injections of the same sample, while six sample solutions were analyzed to determine repeatability. Stability studies on the sample solution were performed at 0, 2, 4, 6, 12, 18, and 24 h. The RSDs of precision, repeatability, and sample stability were calculated in terms of the compound relative peak area and were less than 3%, indicating that the established method was reliable and reproducible and that the sample solution could be stable at room temperature for 24 h. 

### 2.2. Analysis of Inhibitory Activity of P. lobata Extract on PTP1B

**Figure 3:**
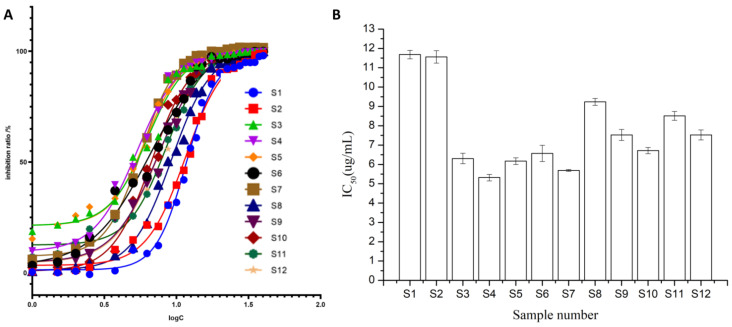
The inhibitory activity of 12 batches of *P. lobata* samples on PTP1B. (**A**), Dose–effect curve; (**B**), IC_50_.

The inhibition ratio and IC_50_ were employed to evaluate the inhibitory activity of *P. lobata* extract on PTP1B. The dose–effect curve was drawn using the logarithm value of sample concentration as the abscissa and the inhibition ratio as the ordinate. The results in Figure 3A demonstrated a clear dose-dependent relationship between the inhibition ratio and the concentration, with higher concentrations resulting in greater inhibition activity. As shown in Figure 3B, the IC_50_ ranged between 5.318 ± 0.17 μg/mL and 11.68 ± 0.22 μg/mL, indicating that the *P. lobata* samples exhibited inhibitory activity against PTP1B. Since the IC_50_ was negatively correlated with the inhibitory activity of PTP1B, and S4 and S7 exhibited higher inhibitory activity than other batch samples, with IC_50_ values of 5.318 ± 0.17 μg/mL and 5.690 ± 0.044 μg/mL, respectively. The IC_50_ of S1 was 2.2 times that of S4, showing a significant difference in inhibitory activities against PTP1B between different batches of samples.

### 2.3. Multivariate Analysis Results

#### 2.3.1. Grey Relational Analysis (GRA)

Grey relational theory, a method used widely in many fields, was first proposed by Deng in his 1982 paper and was designed to analyze uncertain and incomplete information systems with limited collected data. The grey relational grade (GRD) indicates the relational grade between each compared series and the reference series. In this study, IC_50_ was chosen as the reference series, and the peak areas of the 60 common peaks were regarded as the compared series. The grey relational coefficient between the compared and reference series was calculated at a resolution rate of 0.5, and GRD reflected the inhibitory activity of PTP1B.

**Figure 4:**
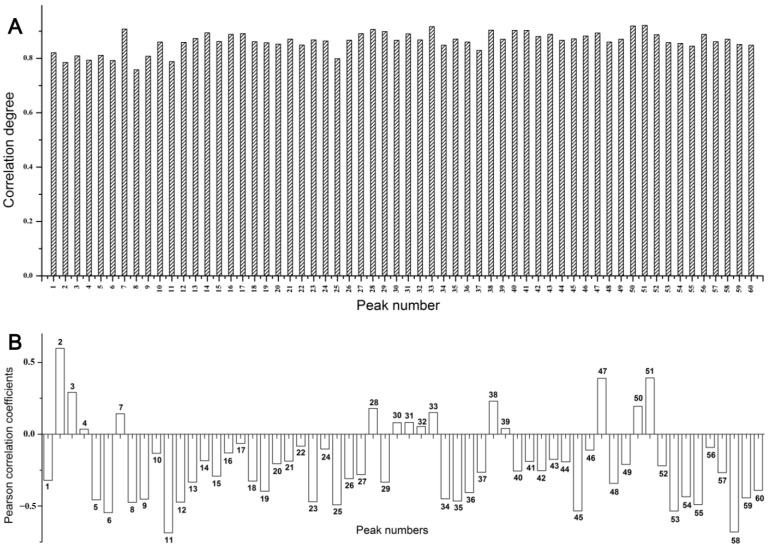
The grey relational grade (**A**) and the Pearson correlation coefficients (**B**) between the peak area and inhibitory activity of PTP1B (IC_50_).

The results of GRA are shown in Figure 4A, and the contributions of the common peaks to the inhibitory activity against PTP1B are shown in Table S2. The GRD between the relative contents of the 60 common peaks and the inhibitory activity of PTP1B were in the range of 0.758–0.921. The GRD values of all the peaks were greater than 0.6, suggesting that the common peak areas were correlated with IC_50_; thus, all peaks demonstrated an important effect on the inhibitory activity of *P. lobata*. Moreover, the GRD values of the peaks, except for 2, 4, 6, 8, 11, and 25, were greater than 0.8, demonstrating the strong correlation between common peak area and inhibitory activity. 

#### 2.3.2. Bivariate Correlation Analysis (BCA)

A BCA was performed using Pearson’s correlation analysis. The peak areas of the common peaks were considered as a group of variables, and the drug efficacy index, IC_50_, was taken as another group of variables. Pearson’s correlation coefficient was used as an index to analyze the correlation size, significance, and direction of change between the two variables. The results (Figure 4B) showed that the peak areas of 48 among the 60 common peaks were negatively correlated with IC_50_.; That is, with an increase in peak area, the inhibitory activity of the extracts on PTP1B increased. The absolute values of correlation coefficients of peaks 5 (puerarin 6″-*O*-xyloside), 6, 8, 9 (glycitin), 11 (genistin), 12 (4′-methoxypuerarin), 23, 25, 34, 35, 36, 45, 53, 54, 55, 58, and 59 were all greater than 0.4, indicating a good correlation. As shown in Table S3, peaks 5 and 6 showed a significant negative correlation (*p* ≤ 0.05). 

#### 2.3.3. Hierarchical Cluster Analysis (HCA)

**Figure 5:**
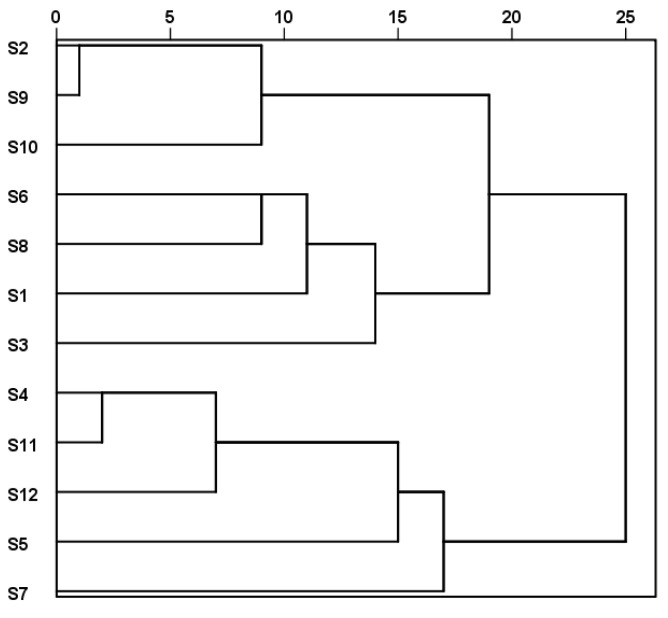
The clustering analysis tree diagram of 12 batches of *P. lobata*.

Cluster analysis is a multivariate statistical method used to classify research subjects into relatively homogeneous categories. In our HCA, the peak areas of the 60 common peaks were used as variables. The variables were standardized by Z-score. The distance was measured by cosine, and the within-groups linkage method was used for cluster analysis. A dendrogram is shown in Figure 5. When the classification distance was 19, 12 batches of *P. lobata* were divided into two categories. Samples S1, S2, S3, S6, S8, S9, and S10, collected from Anhui Province, were clustered into one category, and S4, S5, S7, S11, and S12, collected from Hebei Province, were clustered into another. The results of cluster analysis were consistent with those of the origin of the samples.

#### 2.3.4. Partial Least Squares Discriminant Analysis (PLS-DA)

**Figure 6:**
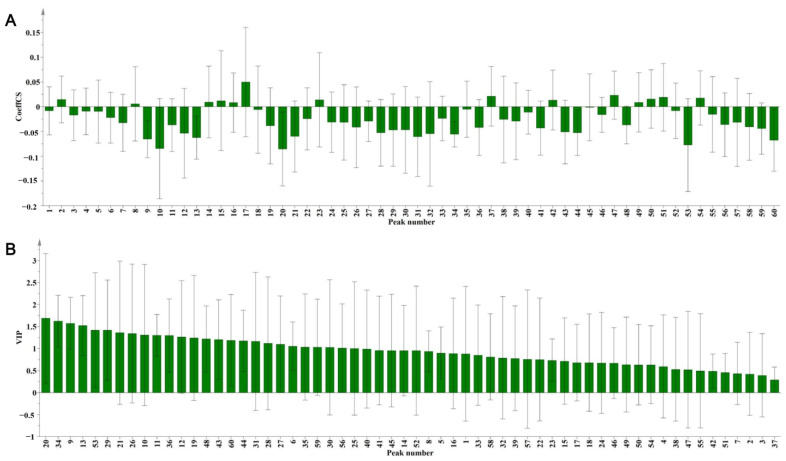
The correlated coefficient (**A**) and the variable importance of projection (VIP) value (**B**) of 60 common peaks of *P. lobata* extracts analysis by PLS-DA.

To reflect the differences between the groups, PLS-DA in the supervised mode was used to identify and screen the components with a large contribution rate to the differences between groups. The PLS-DA model was used to identify the potential active compounds related to the inhibitory activity on PTP1B by correlating the fingerprint chromatographic data and the IC_50_ values. An X matrix of dimensions (12 × 60) from the common peak areas and a Y matrix of IC_50_ values were used. The R^2^X, R^2^Y, and Q^2^ values of the model were 0.587, 0.99, and 0.867, respectively, which were greater than 0.5, indicating good fitting and prediction abilities. This model can be used for the pattern recognition of *P. lobata* from different origins. For these *P. lobata* samples, all peaks, except peaks 2, 8, 14, 15, 16, 17, 23, 37, 42, 49, 50, 51, and 54, were negatively correlated with the IC_50_ values (Figure 6A).

Furthermore, we used the variable importance of projection (VIP) parameter to screen for variables responsible for the inhibitory activity on PTP1B. Compounds with variables above the VIP-value threshold of 1.0 were filtered out as candidate bioactive compounds. There were 26 peaks with VIP values greater than 1.0, which were considered candidate active substances (Figure 6B, Table S4).

#### 2.3.5. Integration of Analytical Results

In the comprehensive analysis, 10 common peaks satisfied the following criteria: negative correlation and a VIP value greater than one in the PLS-DA, negative correlation and a correlation coefficient greater than 0.4 in the BCA, and a correlation degree greater than 0.6 in the GCA. Based on this, peaks 6, 9 (glycitin), 11 (genistin), 12 (4′-methoxypuerarin), 25, 34, 35, 36, 53, and 59 were preliminarily identified as candidates with PTP1B inhibitory activity.

### 2.4. Verification Experiment of PTP1B Inhibitory Activity

According to the results of the spectrum activity relationship analysis, glycitin, genistin, and 4′-methoxypuerarin were identified as the active substances in *P. lobata* inhibiting PTP1B, and the activity was verified using standard samples. Furthermore, chlorogenic acid has been reported to have inhibitory activity against PTP1B [36], so it was selected as a positive control in this study. In addition, the inhibition of oleanolic acid activity against PTP1B was determined. Glycitin, genistin, 4′-methoxypuerarin, chlorogenic acid, and oleanolic acid were diluted in 10% dimethyl sulfoxide (DMSO) Tris-HCL to determine the inhibitory activity of single compounds on PTP1B. The IC_50_ values of glycitin, genistin, 4′-methoxypuerarin, and chlorogenic acid were 10.56 ± 0.42 μg/mL, 16.46 ± 0.29 μg/mL, 9.336 ± 0.56 μg/mL, and 15.28 ± 0.35 μg/mL, respectively, while oleanolic acid had no inhibitory activity against PTP1B. Therefore, oleanolic acid was employed as a negative control for the subsequent experiment.

### 2.5. Molecular Docking 

The crystal structure of PTP1B (1XBO) was downloaded from the RCSB Protein Data Bank (http://www.pdb.org/ (accessed on 1 July 2023)) and was modified using a protein preparation procedure. The receptor grid was generated by removing ligands and water, adding hydrogen, optimizing amino acids, and refining. The ligand structures of glycitin (PubChem CID: 187808), genistin (PubChem CID: 5281377), 4′-methoxypuerarin (PubChem CID: 5319486), chlorogenic acid (positive control) (PubChem CID: 1794427), and oleanolic acid (negative control) (PubChem CID: 10494) were downloaded from the National Library of Medicine (https://pubchem.ncbi.nlm.nih.gov/ (accessed on 8 January 2024)). The ligPrep procedure was used to create the 3D chemical structures with minimal energy. The ligand and receptor grids were then imported into the ligand-docking procedure for calculation and visualization. 

**Figure 7:**
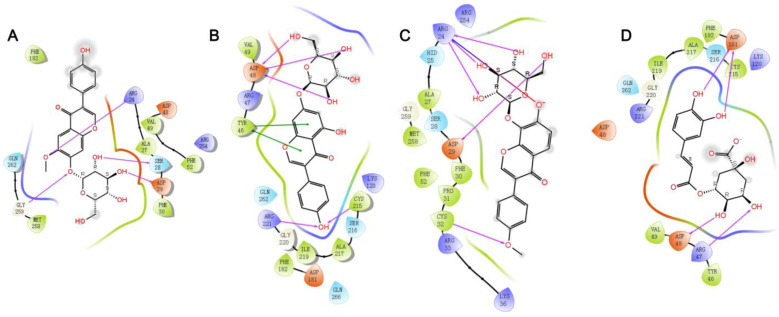
Ligand–protein interaction diagrams. (**A**), Glycitin; (**B**), genistin; (**C**), 4′-Methoxypuerarin; (**D**), chlorogenic acid; 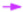 represent hydrogen bonds; 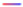 represent salt bridge; 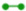 represent π–π stacking.

As shown in Figure 7, four ligands (glycitin, genistin, 4′-methoxypuerarin, and chlorogenic acid) and the PTP1B protein showed good binding activity, and the molecules were connected by hydrogen bond and π bond, whereas oleanolic acid (negative control) failed to dock. This validates the reliability of the molecular docking model. Moreover, glycitin formed hydrogen bonds with the amino acid residues GLY259, SER28, ASP29, and ARG24 of the protein. Genistin formed hydrogen bonds with the amino acid residues ASP48, CYS215, and ARG221 of the protein, and π bonds with TYR46. 4′-methoxypuerarin formed hydrogen bonds with the amino acid residues ASP29, ARG24, and CYS32 and a salt bridge with ARG24 of the protein. Chlorogenic acid formed hydrogen bonds with the amino acid residues ASP181, ASP48, and ARG47 of the protein. The docking scores were −3.908, −5.414, −4.018, and −5.369, respectively.

## 3. Materials and Methods

### 3.1. Reagents and Materials

Reference standards of 3′-hydroxypuerarin (DST220316-075), puerarin 6″-*O*-xyloside (DST200912-054), 3′-methoxypuerarin (DST211203-224), glycitin (DST201129-008), genistin (DST 210319-003), 4′-methoxypuerarin (DST200425-310), ononin (DST211218-044), glycitein (DSTDH000901), genistein (DSTDR000201), formononetin (DSTDM001102), and biochanin A (DSTDY010901) were obtained from Chengdu Desite Bio-Technology Co., Ltd. (Chengdu, China). Daidzein (20211031) was obtained from Shandong Xiya Chemical Co., Ltd. (Linyi, China). PTP1B was obtained from Zoonbio Biotechnology Co., Ltd. (Nanjing, China). HPLC-grade methanol was obtained from Shanghai CINC High Purity Solvent Co., Ltd. (Shanghai, China), and formic acid was obtained from Tianjin Kemiou Chemical Reagent Co., Ltd. (Tianjin, China). Analytical grade reagents, including Tris, anhydrous sodium carbonate, ethanol, DMSO, and chloroform, were obtained from Sinopharm Chemical Reagent Co., Ltd. (Shanghai, China). 4-Nitrophenyl phosphate disodium salt hexahydrate (PNPP) was obtained from Shanghai Macklin Biochemical Technology Co., Ltd. (Shanghai, China).

Twelve batches of samples were collected from different regions of China. The details of the plant samples are presented in Table 1.

### 3.2. Sample Preparation

*P. lobata* samples were crushed and sifted through a 60-mesh sieve. The powders (10 g) were added to flasks containing 100 mL of chloroform, and reflux was performed three times for 2 h at a time. The resulting solution was evaporated under reduced pressure. *P. lobata* extract was obtained by vacuum drying.

Approximately 10 mg of *P. lobata* extract was diluted with DMSO to obtain a 10 mg/mL stock solution. On one hand, 1 mL of the stock solution was then diluted to 10 mL with methanol for HPLC analysis. On the other hand, the sample solution for activity analyses was prepared by diluting the stock solution to a concentration of 1–40 μg/mL with a 10% DMSO Tris-HCl (10 mM, pH 7.35) solution. All solutions were filtered through a 0.22 μm syringe filter before testing.

### 3.3. HPLC Analysis

The fingerprints of the *P. lobata* extract were performed using the Shimadzu LC-20A ultra-fast liquid chromatography (UFLC) system equipped with a UV detector (Shimadzu, Kyoto, Japan). A Tskgel ODS-100V C18 column (4.6 mm× 250 mm, 3 μm) (Tosoh) was employed to separate the extract at 40 °C. As mobile phase at the flow rate of 1.0 mL/min, 0.1% formic acid aqueous (A) and methanol (B) were used, and the optimized gradient elution procedure was as follows: 0–8 min, 22% B; 30–50 min, 45%–60% B; 70–100 min, 70–90% B; 110–115 min, 90–22% B. The detection wavelength was set at 254 nm, and 20 μL of the sample were injected into the HPLC system.

### 3.4. Determination of Inhibitory Activity of P. lobata on PTP1B In Vitro

The inhibitory activity on PTP1B was evaluated using a modified procedure. Briefly, a 12.5 μg/mL PTP1B solution was prepared by diluting 50 μL PTP1B stock solution (0.5 mg/mL) with Tris-HCl (10 mM, pH 7.35) to 2 mL. Simultaneously, a PNPP solution was prepared by dissolving 10 mg of PNPP in 20 mL water, resulting in a concentration of 0.5mg/mL as the substrate solution. Subsequently, a test solution was prepared by combining 100 μL of the sample solution (1–40 μg/mL), 100 μL of the PNPP solution, 180 μL of 10% DMSO Tris-HCl (10 mM, pH 7.35), and 20 μL of the PTP1B solution. The test solution was incubated at 37 °C for 30 min, followed by the immediate addition of a 200 μL sodium carbonate solution (1M) to terminate the reaction. Subsequently, 200 μL of the resulting solution were transferred to a well in a 96-well plate, and the absorbance (A1) was measured at 405 nm using a SynergyH1 Hybrid Multi-Mode Microplate Reader (BioTek, Winoski, VT, USA).

By employing the same methodology, the absorbance (A2) of the blank solution was determined when 100 μL of 10% DMSO Tris-HCl (10 mM, pH 7.35) was used to replace the sample solution, and another 100 μL of Tris-HCl (10 mM, pH 7.35) was used instead of the PTP1B solution. Additionally, the absorbance (A0) of the normal solution was obtained by replacing the sample solution with 100 μL of 10% DMSO Tris-HCl (10 mM, pH 7.35). The inhibition ratio (%) was calculated by the following formula:

$$Inhibition\left(\%\right)=\frac{A0-A1}{A0-A2}\times 100\%$$

### 3.5. Multivariate Analysis

The HPLC fingerprints generated using 12 batches of *P. lobata* samples were assessed using the Similarity Evaluation System for Chromatographic Fingerprint of TCM (2013.130723 Version, Committee of Chinese Pharmacopoeia). Specifically, the data files of 12 batches of *P. lobata* generated by HPLC analysis were initially exported into AIA format files, which were then imported into the Similarity Evaluation System for Chromatographic Fingerprint of TCM software (2013.130723 Version). In the software, the median method was adopted, the time window width was set at 0.2, multi-point correction was carried out, and the full chromatogram peak matching was performed. Finally, the reference chromatogram was generated using sample S4 as the reference.

GraphPad Prism 9 (GraphPad Software, San Diego, CA, USA) was used to draw the dose–effect curve and calculate the IC_50_ value. The dose–effect curve was plotted with the logarithm value of the sample concentration as the *X*-axis and the inhibition rate against PTP1B as the *Y*-axis. The IC_50_ value was then calculated using the dose-response-inhibition equation in the non-linear regression model.

SPSS 25.0 statistical software (SPSS Inc, Chicago, IL, USA) was used to perform BCA and HCA. The peak areas of the common peaks and IC_50_ values for each sample were input into SPSS 25.0 software for analysis. For BCA, the Pearson correlation coefficient was utilized to examine the relationship between the peak area and the IC_50_ value. When HCA was carried out on the data, the peak area was taken as the variable, and the Z-score was used to standardize the data. Subsequently, cosine values were employed to measure distances, and a tree graph was generated through the within-groups clustering method.

SIMCA 14.1 (Umetrics, Umea, Sweden) was used for PLS-DA. The peak areas and IC_50_ values of the common peaks in each sample were inputted into SIMCA 14.1 software. Homologous samples were grouped together, and the confidence level was set at 95%. The coefficients were calculated using the scaled and centered method, and the UV method was used for scaling.

SPSS Pro software (www.spsspro.com (accessed on 8 July 2023)) was used for GRA. The feature sequence was determined as the peak area of the common peaks in each sample, while the parent sequence was defined as IC_50_. The distinguishing coefficient ρ was set to 0.5. The data were standardized prior to analysis.

## 4. Conclusions 

In this study, an HPLC fingerprint of *P. lobata* extract was established, revealing 60 common peaks. The inhibitory activity of *P. lobata* extract on PTP1B was investigated in vitro, considering the crucial role of PTP1B in insulin regulation. The results of the pharmacological activity tests showed that the chloroform extract of *P. lobata* had a significant inhibitory effect on PTP1B. Based on multivariate analyses, 10 common peaks met the set requirements simultaneously and were selected as substances with PTP1B inhibitory activity. Three compounds, genistein, glycine, and 4′ -methoxypuerarin, were identified by comparison with reference standards, and they bound to the PTP1B protein through hydrogen, π bonds, and salt bridge. The IC_50_s of these three compounds against PTP1B in vitro were 10.56 ± 0.42 μg/mL, 16.46 ± 0.29 μg/mL, and 9.336 ± 0.56 μg/mL, respectively, demonstrating that the spectrum–effect relationship method is of great significance in screening the active components of TCMs. This study provides a method for screening PTP1B enzyme inhibitors from *P. lobata*, which helps to clarify the material basis of *P. lobata* in the treatment of diabetes. It also provides a reference for TCM to treat diabetes based on new targets and also promotes the discovery of the active components in TCMs that act on new targets.

## Figures and Tables

**Table 1:** The origin and similarity of 12 batches of *P. lobata*.

Sample No.	Origin	Longitude and Latitude	Similarity
S1	Liuan, Anhui Province, China	116.53 east, 32.01 north	0.959
S2	Anqing, Anhui Province, China	116.50 east, 30.20 north	0.955
S3	Tongling, Anhui Province, China	117.61 east, 30.89 north	0.969
S4	Nanyang, Henan Province, China	112.39 east, 32.96 north	0.981
S5	Pingdingshan, Henan Province, China	113.24 east, 33.56 north	0.987
S6	Liuan, Anhui Province, China	116.28 east, 32.06 north	0.966
S7	Luohe, Henan Province, China	113.77 east, 33.61 north	0.962
S8	Huaibei, Anhui Province, China	116.71 east, 33.91 north	0.963
S9	Fuyang, Anhui Province, China	116.13 east, 32.75 north	0.978
S10	Anqing, Anhui Province, China	116.26 east, 30.31 north	0.961
S11	Xinyang, Henan Province, China	114.50 east, 31.83 north	0.888
S12	Nanyang, Henan Province, China	112.77 east, 32.90 north	0.946

## Data Availability

The data presented in this study are available on request from the corresponding author.

## Supplementary Materials

The following supporting information can be downloaded at https://www.mdpi.com/article/10.3390/molecules29122731/s1: Figure S1: HPLC chromatograms of reference fingerprint and reference standards; Figure S2: Molecular docking diagram; Table S1: Retention time and peak area of common peaks of 12 batches of *P. lobata*; Table S2: GRA results of common peaks on PTP1B inhibitory activity; Table S3: BCA results of common peaks on PTP1B inhibitory activity; Table S4: VIP value of the common peaks in PLS-DA.
