# Supplementary material for: Protein Tyrosine Phosphatase 1B Inhibitors of Pueraria lobata Based on the Spectrum–Effect Relationship by Q-Marker Selection
Source: Molecules. 2024 Jun 8;29(12):2731. doi: 10.3390/molecules29122731 (PMC11207073; doi:10.3390/molecules29122731)
Supplement: Supplementary file 1 [file molecules-29-02731-s001.zip › molecules-3012286-supplementary.pdf]

Electronic Supplementary Information

Protein Tyrosine Phosphatase 1B inhibitors of *Pueraria lobata* based on the spectrum-effect relationship by Q-marker selection

Yong Zhang [1], Haipeng Liu [2], Tianci Lv [2], Mengqian Xiao [2] and Guihua Gao [1*]

[1] *School of Pharmacy, Jining Medical University, Rizhao 276826, P. R. China;*

[2] *School of Pharmacy, Shandong University of Traditional Chinese Medicine, Jinan 250355, P. R.China*

1

[*]**Correspondence:** School of Pharmacy, Jining Medical University, Rizhao, Shandong Province, 276826, P. R. China

E-mail address:guihua526@163.com

Fax: +86-633-2983690

Content List

1. HPLC chromatograms of reference fingerprint and reference standards (Figure S1), Page 3

2. Molecular docking diagram (Figure S2), Page 4

3. Retention time and peak area of common peaks of 12 batches of *P. lobata* (Table S1), Page 5

4. GRA results of common peaks on PTP1B inhibitory activity (Table S2), Page 8

5. BCA results of common peaks on PTP1B inhibitory activity (Table S3), Page 9

6. VIP value of the common peaks in PLS-DA (Table S4), Page 10

1. HPLC chromatograms of reference fingerprint and reference standards

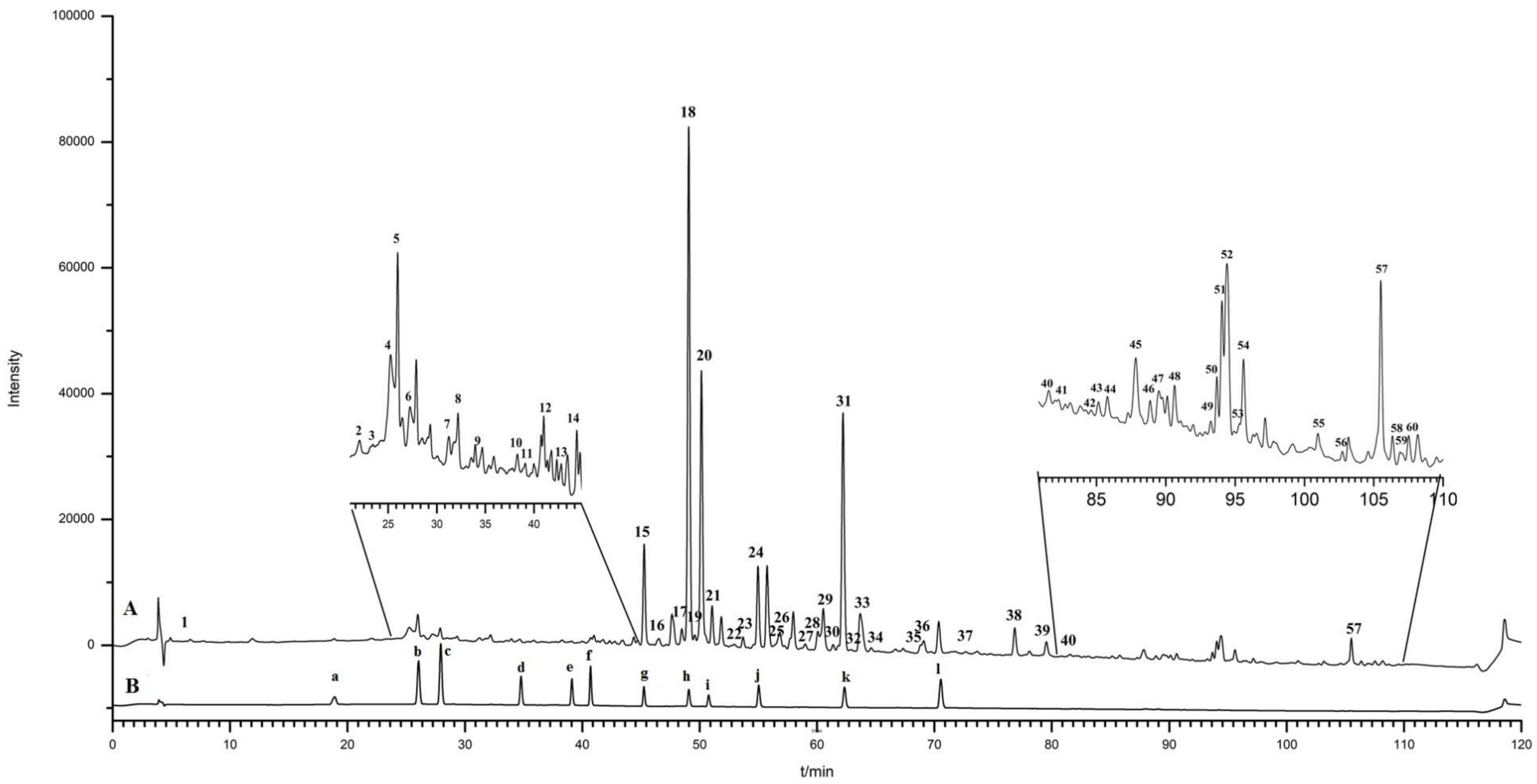

Figure S1 HPLC chromatograms of reference fingerprint (a) and reference standards (b). a, 3'-Hydroxypuerarin; b, Puerarin 6''-O-xyloside; c, 3`- Methoxy Puerarin; d, Glycitin; e, Genistin; f, 4'-Methoxypuerarin; g, Ononin; h, Daidzein; i, Glycitein; j, Genistein; k, Formononetin; l, Biochanin A

2. Molecular docking diagram

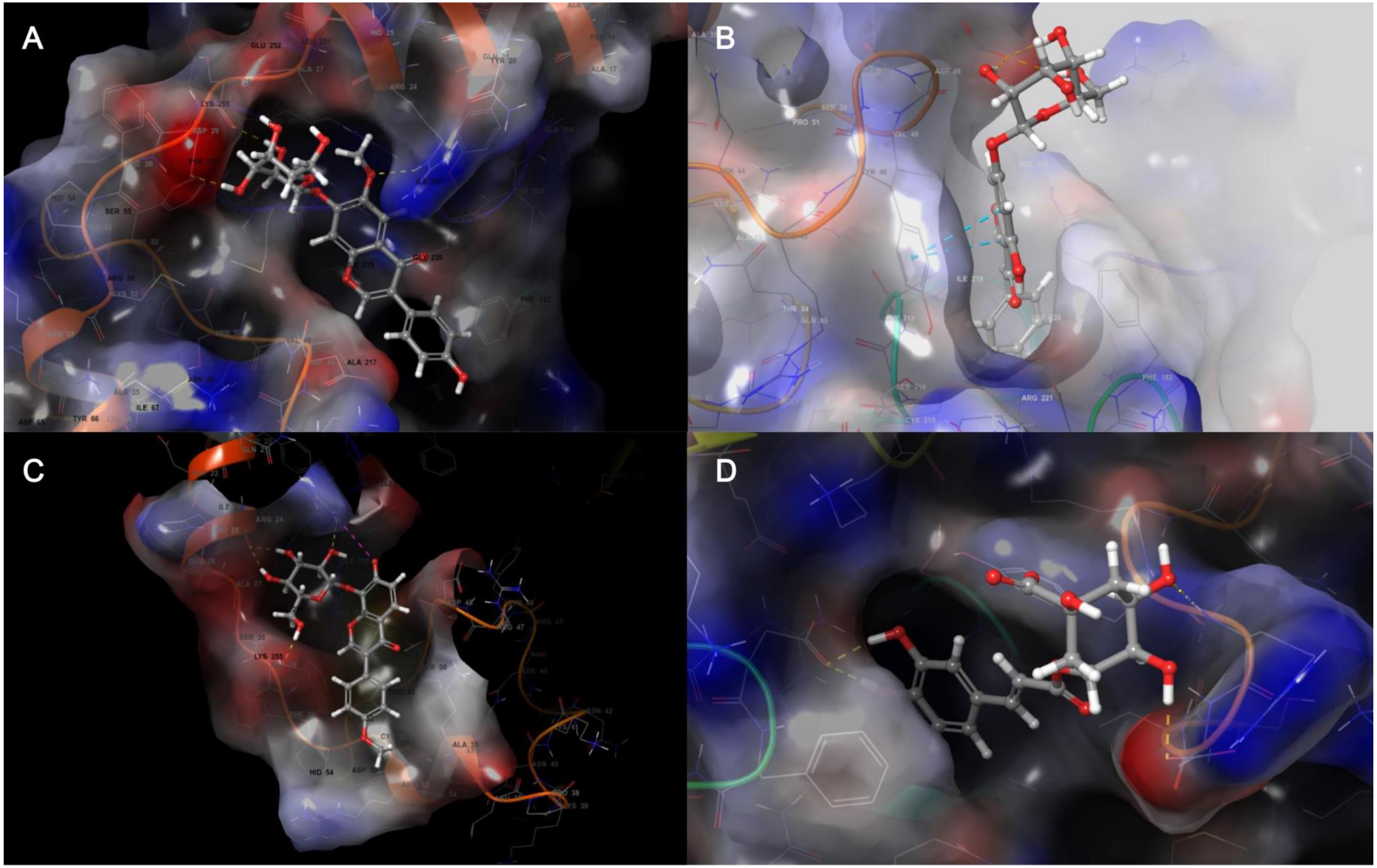

Figure S2 Molecular docking diagram of the active component and PTP1B. A, Glycitin; B, Genistin; C, 4'-Methoxypuerarin; D, Chlorogenic acid

3. Retention time and peak area of common peaks of 12 batches of *P. lobata*

Table S1. Retention time and peak area of common peaks of 12 batches of *P. lobata*.

**NO.**	Rt (min)	S1	S2	S3	S4	S5	S6	S7	S8	S9	S10	S11	S12	Area RSD(%)	Rt RSD(%)
1	5.95	4882.7	3522.5	5720.2	4523.9	5801.6	3780.6	44381.1	3943.2	3837.5	3560.8	3663.5	5122.4	149.7	0.53
2	20.378	160536.8	24379.0	8175.2	11397.8	9207.6	6349.5	28538.1	4410.2	4923.2	4448.3	9678.7	7156.0	188.8	0.36
3	21.83	45796.9	2575.7	2422.3	13273.0	10594.7	3360.2	24866.4	3719.2	4223.7	7796.0	9714.3	12439.8	106.5	0.26
4	23.255	30882.2	1015.5	9837.4	15264.3	7564.7	2472.7	29784.2	6757.6	1506.9	2598.4	4754.5	8020.2	102.6	0.26
5	24.712	58001.0	14141.9	88983.2	105028.6	40103.8	34530.9	78896.2	13270.1	9896.5	13136.0	29114.1	55551.9	72.0	0.21
6	26.642	13318.5	9253.5	29154.4	35893.5	24958.1	4318.2	38518.6	13216.8	5428.6	9971.2	5250.7	19454.7	69.4	0.17
7	29.582	32927.8	18014.1	13085.3	19827.3	23206.7	27684.8	19383.2	14002.1	24030.2	30191.9	28075.4	35033.9	30.1	0.18
8	30.372	3016.9	2782.2	13239.8	11276.8	2040.6	2362.9	12529.8	2755.2	1133.6	2528.9	2548.4	11938.9	86.1	0.23
9	34.339	3947.9	7850.9	6409.9	12951.1	20845.6	4588.9	24960.4	6925.6	6996.5	17660.5	19855.4	22560.1	59.8	0.13
10	36.908	10537.3	4801.3	8396.6	9428.8	17631.0	13255.2	7138.9	9106.5	5250.0	3427.3	15281.3	17331.4	47.5	0.1
11	37.945	1362.0	2189.5	6105.4	7904.8	6478.0	1766.1	7946.2	1935.4	1278.3	2641.3	2186.6	5138.0	66.6	0.16
12	39.954	20005.7	11163.9	29950.8	24051.0	40750.1	14141.5	33013.7	23226.8	9671.3	19331.3	22232.6	30967.2	40.0	0.12
13	42.524	10757.6	16581.0	12160.3	20887.2	18232.2	10955.7	30896.9	11490.1	20024.3	14794.6	27117.9	24159.5	36.7	0.08
14	43.48	12490.1	16349.3	18235.7	12190.5	14473.1	8246.6	25312.8	13512.9	12322.6	18103.6	16824.1	19827.3	28.5	0.05
15	44.329	142279.0	164982.0	218241.3	150777.2	233936.0	77794.4	248917.5	149699.5	78165.7	227037.8	142004.2	141047.1	34.6	0.06
16	45.486	22401.7	18251.7	20322.7	16307.2	12747.6	11719.9	42699.3	7703.7	12939.7	12999.3	15732.7	18603.1	50.1	0.07
17	47.463	33293.5	36046.9	46851.7	24560.1	38446.4	23451.5	44389.4	30996.1	19646.8	35252.0	25937.0	23536.5	27.3	0.13
18	48.004	437284.8	397655.6	1146771.3	1217339.4	500276.9	671312.8	365490.8	659475.6	247178.6	337754.8	947074.6	636794.8	50.7	0.06
19	48.549	13193.0	19675.9	23183.8	35992.2	14757.5	25420.7	26081.7	10515.8	17030.9	11940.2	33005.4	29552.7	39.2	0.07
20	49.069	627434.1	646599.8	648856.3	1458890.3	1115367.0	517431.6	754108.7	695690.3	422734.5	402439.4	1551733.3	1161307.4	47.0	0.07
21	49.978	66312.4	72687.7	92053.1	156849.3	94109.9	56614.4	68252.5	90587.3	39699.6	48585.5	142655.4	105633.5	41.4	0.07
22	51.518	4669.9	7152.7	2044.1	6079.0	4642.9	1390.2	13432.2	3491.7	8535.2	2778.3	2686.9	5437.0	64.6	0.15
23	51.879	5203.4	6051.4	13637.9	10591.4	6994.6	5778.8	7884.0	4143.0	5138.2	4063.6	6768.1	7373.0	39.6	0.1

24	53.808	146815.3	78673.6	199404.8	252469.0	143993.5	130768.3	72267.5	219587.0	48885.5	59629.2	195094.6	149445.7	47.1	0.07
25	54.639	70021.6	66423.3	205681.1	349360.2	85928.0	269826.9	158234.8	64763.7	57428.6	46563.3	258090.7	209171.3	67.0	0.09
26	55.616	26881.4	67287.8	59673.0	104257.1	45656.2	56043.4	69554.2	30622.3	40474.3	42092.6	107492.5	80766.1	43.3	0.14
27	56.105	5123.8	9806.1	6918.8	8417.1	9894.0	4055.7	16524.2	7288.6	7073.5	7082.7	6824.8	8954.7	38.5	0.08
28	58.748	57270.6	44227.4	45983.0	62474.5	50716.2	36033.1	32906.9	46842.2	31569.4	41476.3	80341.9	63756.8	29.1	0.08
29	59.196	138309.1	93485.4	129281.4	189559.9	106117.3	105439.7	178641.4	96807.1	116196.1	114579.7	183396.5	170393.6	26.6	0.08
30	60.023	12312.0	62751.6	12983.2	60597.9	33614.2	14297.5	24983.5	21846.6	27413.4	31146.8	59271.6	45398.0	55.5	0.08
31	60.849	667447.2	584264.4	584012.1	1034009.9	719861.3	481570.1	452022.1	858640.8	289182.5	410555.8	1200676.1	764646.9	39.5	0.08
32	61.57	16934.1	21162.8	2428.0	16420.2	21222.7	11315.4	21600.0	6502.8	20977.9	12762.6	8213.1	21320.3	44.6	0.11
33	62.36	216647.0	135730.6	147499.6	134936.3	142373.8	118163.7	245623.6	149766.7	113541.7	108497.9	162896.7	197841.0	27.4	0.13
34	63.272	6936.1	10379.7	10146.6	17229.4	15259.6	4422.8	23173.7	2948.8	10327.4	10932.8	18501.1	15746.9	49.2	0.09
35	67.381	21642.7	25586.9	44712.3	33957.6	26491.7	23157.1	82703.9	16213.9	37229.5	25752.3	36749.6	40532.1	50.3	0.1
36	67.716	6429.3	6362.4	8073.0	12898.6	6273.4	7393.9	22032.0	6283.5	11173.6	5950.9	14237.4	14202.8	49.1	0.1
37	71.979	14840.1	22266.1	11277.8	30913.1	27428.3	11033.2	49858.9	6205.1	40898.8	139933.1	40578.3	29875.1	100.5	0.1
38	75.32	149097.7	111313.4	71743.3	81900.8	107381.0	91877.6	143794.0	67146.5	89041.3	56757.4	53617.6	141148.5	34.7	0.11
39	77.984	71815.9	154043.0	37952.7	80313.9	120090.8	46830.1	152696.0	31008.9	96368.2	86456.6	67396.0	108552.3	46.4	0.11
40	79.982	11674.9	11788.9	9950.3	11677.2	11881.1	8956.5	21777.4	8442.5	9703.2	12364.4	10868.9	10924.2	29.3	0.12
41	81.287	3939.0	5095.5	3586.4	4393.8	6218.2	4108.4	11858.0	7722.1	5580.4	4226.2	5865.8	6526.8	39.6	0.12
42	83.698	4636.6	2137.8	5188.1	2547.1	2778.5	5564.6	8748.3	4435.6	4460.4	2318.3	2862.0	4326.9	44.7	0.11
43	84.239	11059.1	7408.9	7069.4	8305.5	12572.4	5245.5	14234.9	4104.4	7309.8	7282.2	7432.4	11097.8	35.1	0.1
44	84.959	1699.5	3048.2	1224.4	1839.6	4762.6	1519.5	6188.0	2322.5	2049.8	2642.5	4008.8	2308.9	52.9	0.12
45	86.127	15505.7	14394.3	30769.5	26221.9	17702.0	25365.5	50823.2	19266.2	14279.2	17030.1	25703.7	20929.5	44.1	0.1
46	86.74	1842.6	2918.6	1626.6	2696.1	1913.2	1244.6	4482.6	3079.8	3251.2	3877.5	2818.2	4150.3	36.4	0.1
47	88.416	23253.7	5523.4	11048.1	6459.6	5639.1	15194.6	11636.7	15664.5	5121.5	10754.4	10884.0	13458.1	47.3	0.06
48	89.108	13397.6	23383.1	14912.8	18401.9	31154.8	15423.4	36591.8	2247.4	16539.9	29002.0	29400.5	26547.5	44.9	0.09
49	91.087	2322.8	4527.6	2039.7	3197.6	2727.1	2905.9	8909.5	2770.6	3880.0	7501.1	5545.7	3169.2	52.3	0.12
50	91.807	28885.5	22313.3	19771.8	14640.1	14648.6	11379.3	42063.7	22885.0	9742.4	15674.1	17489.7	17447.7	44.4	0.09
51	92.552	163748.3	104851.5	87336.4	83384.9	60836.8	80464.8	173373.2	146868.7	59047.4	81207.3	88411.7	87740.9	38.0	0.1

52	93.472	15378.7	16060.9	15034.1	18147.2	12932.1	8896.2	49121.9	17350.7	9362.3	14479.3	18137.3	14408.6	59.7	0.09
53	94.019	18889.6	14825.5	29310.6	29084.8	65433.4	31962.3	43149.3	24417.1	14115.9	22971.1	36331.4	37434.9	46.2	0.08
54	94.925	5133.8	11201.6	7057.1	11031.6	6900.6	10960.1	18582.6	1594.4	8687.2	17567.8	6678.4	11132.6	50.1	0.08
55	99.521	6306.3	3738.4	7119.1	6753.7	8019.7	9255.9	5945.4	1596.9	4035.8	6589.2	3626.5	9128.4	39.1	0.07
56	101.281	5156.6	7439.2	3319.5	4437.3	7125.9	5604.9	8813.6	1112.8	4599.2	5939.5	6075.6	8580.3	38.6	0.07
57	104.212	60897.3	15849.3	63236.2	42391.1	68514.0	54686.6	34110.4	13873.3	21698.1	37235.2	43885.6	83419.0	48.6	0.06
58	105.709	5405.8	9088.2	14816.4	14715.7	28282.9	15836.5	22483.3	11667.5	21691.2	26954.7	21216.4	17076.8	39.8	0.1
59	106.269	7539.3	7521.8	11006.5	19758.1	21810.7	6475.2	15844.2	14026.0	11866.1	28104.6	24708.9	15962.9	45.8	0.06
60	106.91	9442.2	8512.2	11404.5	24089.5	34684.9	11262.5	18055.2	25302.4	7111.3	28458.5	27295.1	20287.9	48.9	0.06

4. GRA results of common peaks on PTP1B inhibitory activity

Table S2 GRA results of common peaks on PTP1B inhibitory activity

Peak number	GRD	Rank	Peak number	GRD	Rank	Peak number	GRD	Rank
51	0.921	1	13	0.873	21	53	0.858	41
50	0.919	2	45	0.872	22	19	0.857	42
33	0.917	3	35	0.871	23	54	0.855	43
7	0.908	4	21	0.871	24	20	0.852	44
28	0.906	5	49	0.87	25	59	0.851	45
38	0.904	6	58	0.87	26	22	0.849	46
40	0.903	7	39	0.87	27	60	0.848	47
41	0.903	8	32	0.868	28	34	0.848	48
29	0.898	9	23	0.868	29	55	0.845	49
14	0.894	10	26	0.867	30	37	0.829	50
47	0.893	11	30	0.866	31	1	0.821	51
17	0.891	12	44	0.866	32	5	0.811	52
27	0.891	13	24	0.864	33	3	0.809	53
31	0.89	14	15	0.862	34	9	0.808	54
43	0.888	15	57	0.861	35	25	0.799	55
16	0.888	16	18	0.861	36	4	0.793	56
56	0.888	17	48	0.86	37	6	0.792	57
52	0.887	18	10	0.86	38	11	0.788	58
46	0.882	19	36	0.86	39	2	0.785	59
42	0.88	20	12	0.859	40	8	0.758	60

5. BCA results of common peaks on PTP1B inhibitory activity

Table S3 BCA results of common peaks on PTP1B inhibitory activity

Peak number	Correlation coefficient	Rank	Peak number	Correlation coefficient	Rank	Peak number	Correlation coefficient	Rank
11	-0.686	1	29	-0.334	21	16	-0.13	41
58	-0.68	2	13	-0.334	22	46	-0.111	42
6	-0.546*	3	18	-0.326	23	24	-0.103	43
53	-0.535	4	1	-0.321	24	56	-0.092	44
45	-0.534	5	26	-0.309	25	22	-0.083	45
25	-0.491	6	15	-0.292	26	17	-0.065	46
55	-0.49	7	27	-0.281	27	4	0.035	47
8	-0.474	8	57	-0.267	28	39	0.04	48
12	-0.473	9	37	-0.265	29	32	0.054	49
23	-0.47	10	40	-0.256	30	30	0.08	50
35	-0.465	11	42	-0.254	31	31	0.082	51
5	-0.456*	12	52	-0.22	32	7	0.142	52
9	-0.452	13	49	-0.21	33	33	0.151	53
34	-0.45	14	20	-0.205	34	28	0.18	54
59	-0.443	15	44	-0.192	35	50	0.194	55
54	-0.435	16	41	-0.189	36	38	0.23	56
36	-0.406	17	21	-0.187	37	3	0.291	57
19	-0.397	18	14	-0.185	38	47	0.389	58
60	-0.391	19	43	-0.175	39	51	0.392*	59
48	-0.342	20	10	-0.133	40	2	0.596	60

Note: * $p \leq 0.05$

6. VIP value of the common peaks in PLS-DA

Table S4. VIP value of *P. lobata*.

Rank	Peak number	VIP	Rank	Peak number	VIP
1	20	1.68977	14	48	1.2193
2	34	1.62283	15	43	1.20577
3	9	1.57045	16	60	1.18562
4	13	1.52496	17	44	1.17533
5	53	1.41976	18	31	1.16303
6	29	1.41968	19	28	1.11967
7	21	1.35985	20	27	1.09752
8	26	1.34201	21	6	1.05397
9	10	1.30867	22	35	1.03635
10	11	1.30104	23	59	1.03132
11	36	1.29658	24	30	1.02577
12	12	1.26257	25	56	1.01163
13	19	1.24027	26	25	1.00268
